# Flexural Behavior of T-Shaped UHPC Beams with Varying Longitudinal Reinforcement Ratios

**DOI:** 10.3390/ma14195706

**Published:** 2021-09-30

**Authors:** Rui Zhang, Peng Hu, Kedao Chen, Xi Li, Xiaosen Yang

**Affiliations:** 1 Department of Bridge Engineering, School of Civil Engineering, Southwest Jiaotong University, Chengdu 610031, China; rayz430@swjtu.edu.cn (R.Z.); hupeng123@my.swjtu.edu.cn (P.H.); chen_kedao@my.swjtu.edu.cn (K.C.); 2 Key Laboratory of High-Speed Railway Engineering, Ministry of Education, Southwest Jiaotong University, Chengdu 610031, China; 3 School of Civil Engineering, Qingdao University of Technology, Qingdao 273400, China; 4 Department of Bridge and Tunnel Maintenance, Gansu Transportation Planning, Survey and Design Institute Co., LTD, Lanzhou 730030, China; confidentyou@hotmail.com

**Keywords:** ultra-high-performance concrete (UHPC), flexural behavior, T-shaped beam, reinforcement ratio, theoretical study

## Abstract

In order to investigate the transverse flexural behavior of the UHPC waffle deck, a total of six T-shaped UHPC beams, with varying longitudinal reinforcement ratios, were tested and analyzed. The experiments, including material tests of UHPC and beam tests, were conducted. The material tests of UHPC revealed that strain-hardening behavior in tension was exhibited, and the ratio of uniaxial compressive strength-to-cubic compressive strength was 0.85. The beam tests showed that all the T-shaped UHPC beams, even without longitudinal rebar, exhibited ductile behavior that was similar to that of properly reinforced concrete beams. As the longitudinal reinforcement ratio increased, more flexural cracks developed and a larger load-carrying capacity was provided. Furthermore, the sectional analysis for the ultimate flexural capacity of T-shaped UHPC beams was conducted. Simplified material models, under tension and compression, for UHPC were developed. Based on the reverse calculation from the experimental result, the relation between reduction factor to the ultimate tensile strength of UHPC, and longitudinal reinforcement ratios was formulated. As a result, the predictive equations for the ultimate flexural capacity of T-shaped UHPC beams were proposed, and agreed well with the experimental results in this study and existing studies, which indicates good validity of the proposed equations.

## 1. Introduction

Ultra-high-performance concrete (UHPC), as one kind of emerging fiber-reinforced cementitious composite, characterized by its excellent mechanical performance and durability, was developed in 1970s [1,2,3], and has aroused interest all over the world in recent decades. Different from conventional concrete, UHPC contains a matrix with densely packed particles and a certain volume fraction of steel fibers. Attributing to the low water-to-binder ratio and high compactness, UHPC has extremely low permeability [4], which results in excellent durability. Moreover, UHPC exhibits ultra-high compressive and tensile strengths, up to 150 MPa and 5 MPa, respectively, which are much larger than those of conventional fiber-reinforced concrete (15–80 MPa in compression and 1–3 MPa in tension) [5]. Additionally, UHPC also exhibits strain-hardening behavior under tension, due to the addition of steel fibers [6], which also improves the ductility and energy dissipation capacity of UHPC structural members [7]. Because of all these merits, as aforementioned, using UHPC as bridge deck in the composite bridge has been demonstrated to be a promising way to replace conventional concrete deck.

**Figure 1:**
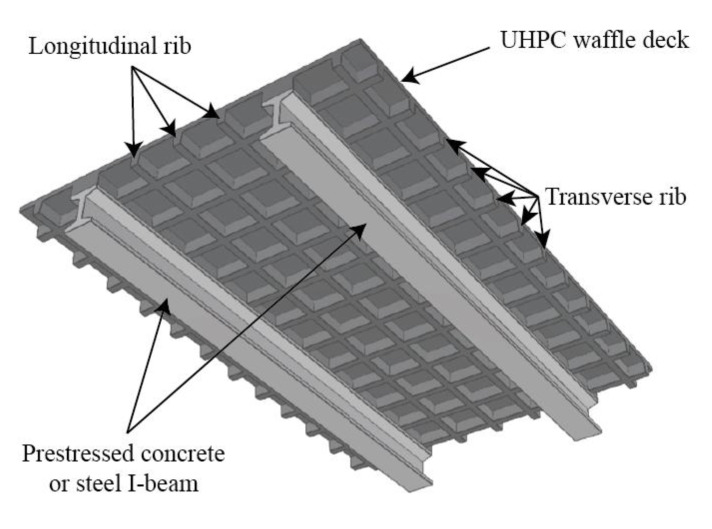
Schematic illustration of UHPC waffle bridge deck.

The high strength of UHPC makes it possible to fabricate thinner and lighter bridge deck with the same capacity as concrete deck. On the other hand, the reduced dimension of the cross section results in the insufficiency of stiffness. Therefore, the concept of UHPC waffle deck was proposed for both new construction and existing bridges [8,9] (Figure 1). The conventional concrete deck is vulnerable to cracking, and then the hazardous substances on the deck penetrate through the cracks and result in the durability problems of concrete deck and steel, or prestressed concrete beams under the deck. Moreover, this technique does not only improve the capacity and durability of the composite bridge, but also realizes rapid construction by precast and assembly, and minimum disruption in traffic. So far, there have been a number of experimental and analytical studies in this field [10,11,12,13,14,15,16,17,18,19,20], with an increasing applications of UHPC waffle deck for new constructions and the replacement of concrete deck in the existing bridges. The field evaluation of the first bridge, using prefabricated UHPC waffle deck, was conducted by Honarvar et al. [21], and the design optimization was suggested. Up to now, the longitudinal flexure [12,13,14,15,16,17,18,20], shear [19], fatigue behavior [10], and connector of steel-UHPC waffle bridge deck [11] have already been reported, but it is rare to see a study on the transverse flexural behavior. In essence, the transverse flexural behavior of UHPC waffle deck could be idealized as the flexural behavior of T-shaped UHPC beams, according to the equivalent strip width [9].

So far, the flexural behavior of steel-reinforced UHPC beams with a rectangular section has been widely studied [22,23,24,25,26,27]. The parameters, including reinforcement ratio [22,23,24], fiber type [25], and content [26,27], were investigated by both experiments and the analysis. However, there are few studies on the flexural behavior of T-shaped UHPC beams. Shao et al. [28,29] studied the flexural behavior of T-shaped UHPC beams by the bending tests and analysis, but the number of specimens were limited, and the effect of longitudinal ratio on the flexural capacity of T-shaped UHPC beams was not fully considered. In summary, previous studies indicated the improvement in the flexural capacity, stiffness, and cracking behavior by using UHPC, but all of them focused on the flexural behavior of UHPC beams with a rectangular section, and few of them proposed predictive equations for flexural capacity with satisfied accuracy. Furthermore, the effect of the longitudinal reinforcement ratio on the flexural capacity of T-shaped UHPC beams was not adequately considered, which resulted in a lack of theoretical basis for the design of UHPC waffle deck in the transverse direction. Therefore, it is necessary to clarify the flexural behavior of T-shaped UHPC beams and propose predictive equations of the flexural capacity of T-shaped UHPC beams with sufficient accuracy, for the design of the transverse direction of UHPC waffle deck.

The purpose of this study was to investigate the flexural behavior of T-shaped UHPC beams with varying longitudinal reinforcement ratios via four-point loading tests. The material model of UHPC was developed based on the compression and uniaxial tensile tests. The relation between the reduction factor to the ultimate tensile strength of UHPC, and the longitudinal reinforcement ratio was formulated. The sectional analysis on the ultimate flexural capacity of T-shaped UHPC beams was conducted, and the predictive equations for flexural capacity were proposed.

## 2. Material Tests

### 2.1. UHPC

**Figure 2:**
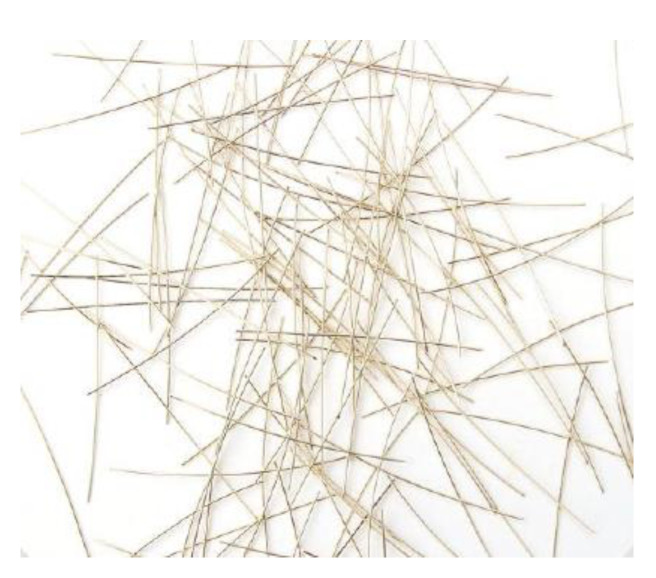
Steel fibers.

The mix of UHPC in this study incorporates water, premixed binder, steel fibers and polycarboxylate superplasticizer. The steel fiber used in this study is shown in Figure 2 and its properties are tabulated in Table 1. Two percent steel fibers in volume fraction of 2% was added. The mix proportion of UHPC, which was a kind of commercial product, is shown in Table 2. UHPC in this study was mixed by a horizontal forced mixer with single shaft. After setting, all UHPC beams and specimens for tensile and compression tests were cured in an environment with a temperature of 20 ± 2 °C and a relative humid above 90% for 28 days.

#### 2.1.1. Compression Tests

Both cube and prism specimens with dimensions of 100 mm × 100 mm × 100 mm and 100 mm × 100 mm × 300 mm, respectively, were prepared to obtain the compressive properties of UHPC. In compression tests, six cubes and six prisms were tested. Table 3 summarizes experimental results of compression tests, in which the mean values of cubic and axial compressive strength are 166.0 and 141.8 MPa, respectively. The ratio between axial and cubic compressive strength was 0.85, which is far greater than that of conventional high-strength concrete [30].

#### 2.1.2. Tensile Tests

**Figure 3:**
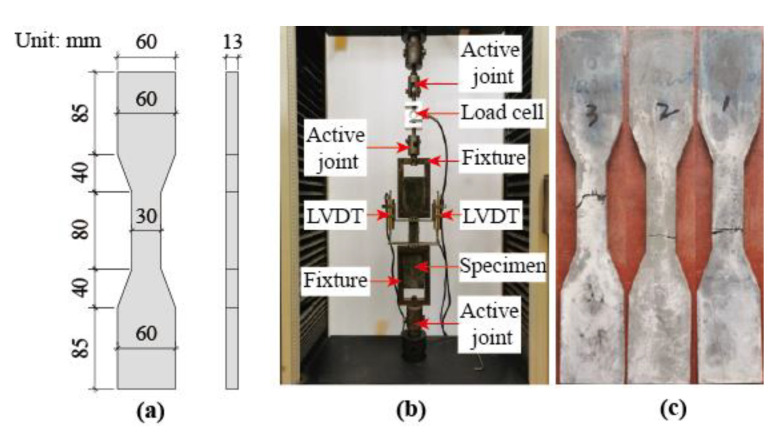
Uniaxial tensile tests of UHPC. (**a**) dimension of specimen; (**b**) test setup; (**c**) tested specimens.

**Figure 4:**
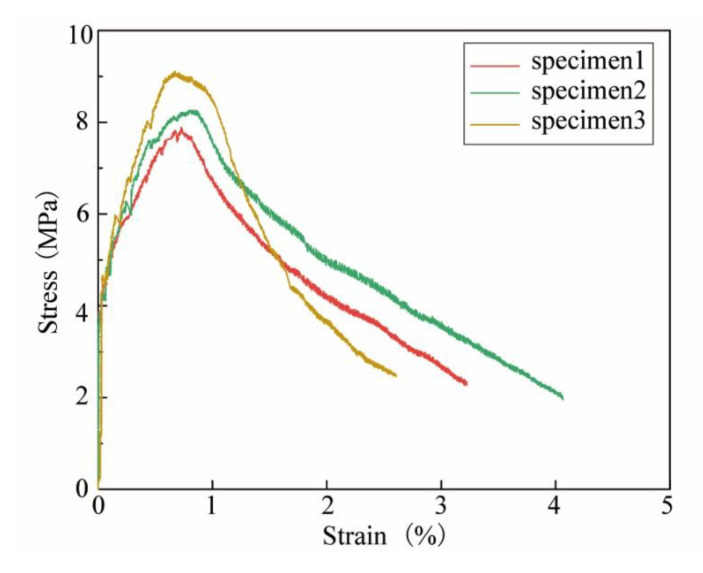
Tensile stress–strain curves.

The uniaxial tensile tests of UHPC were conducted as shown in Figure 3. A total of three specimens with dimensions as shown in Figure 3a were tested. The setup of tensile tests is shown in Figure 3b. A load cell was employed to measure the axial load. Two linear variable differential transformers (LVDT) were used to measure the axial deformation within the gauge length. The tensile load was applied based on the displacement at the speed of 0.1 mm/min. After the first cracking, the tensile stress continued to increase as more cracks developed, whereas the tensile stress began to decrease when the localization of cracks occurred. As a result, the mean value of first cracking, ultimate tensile strength and the strain corresponding to the ultimate strength are 4.14 MPa, 8.42 MPa and 0.007, respectively. Figure 3c shows the damaged UHPC tensile specimens after testing. Figure 4 shows the tensile stress–strain curves of UHPC. The key parameters in these stress–strain curves are tabulated in Table 4.

### 2.2. Rebar

All deformed rebar with a characteristic yield strength of 400 MPa were used in this study. A total of five kinds of diameters of rebar, namely, 6, 10, 12 16, 20 and 22 mm, were used in T-shaped beams. The rebars with diameters of 6 and 10 mm were used as stirrups and steel mesh in flange in all beams, respectively. The rebars with diameters of 6, 12, 16, 20 and 22 mm were employed as tensile longitudinal reinforcement in the web of beams. In tensile tests of rebar, 5 samples at each diameter were prepared. Table 5 summarizes the tensile properties of these rebars based on tensile tests.

## 3. Experimental Program

### 3.1. Design of Beams

**Figure 5:**
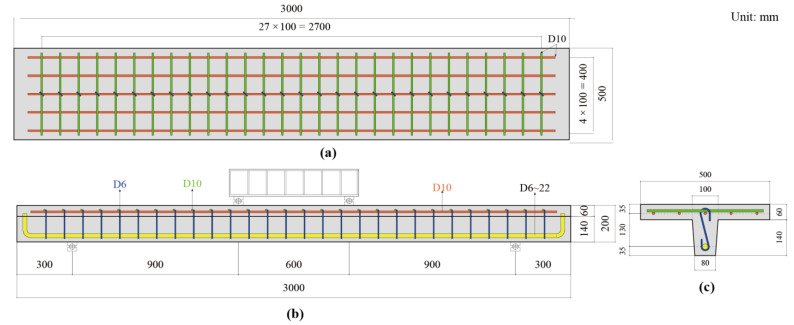
Configuration and dimension of T-shaped UHPC beams. (**a**) plan of flanges; (**b**) elevation; (**c**) cross section.

A total of six T-shaped UHPC beams, as listed in Table 6, including one without longitudinal rebar (UT-00) and five beams with varying longitudinal reinforcement ratio from 0 to 2.04% were prepared for loading tests. ‘UT’ stands for ‘UHPC beam with T-shaped cross section’ and latter two digits stand for the diameter of longitudinal tensile bar. In this study, the calculation of longitudinal reinforcement ratio (*ρ*_l_) for T-shaped beams followed Chinese code [30] and it could be expressed by Equation (1), as follows:

(1) $$\rho_{l}=\frac{A_{s}}{b_{w}h}$$

where *A*_s_ is the total area of longitudinal tensile bars in web (mm); *b*_w_ is the thickness of web (mm); *h* is the total height of T-shaped cross section (mm). The experimental parameter in this study is the longitudinal reinforcement ratio. All beams had the same dimensions and configurations as shown in Figure 5. The total height and width of T-shaped cross section were 200 mm and 500 mm, respectively (*h* = 200 mm, *b*_f_ = 500 mm). The shape of web was not a rectangle, but a trapezoid. The flange was 60 mm thick (*h_f_*′ = 60 mm) and reinforced by the steel bar mesh with a size of 100 mm × 100 mm. The effective height of T-shaped cross section (*h*_0_) was 165 mm in five beams with longitudinal rebar. To prevent potential shear failure, singe-leg stirrups with diameter of 6 mm were arranged with spacing of 100 mm in five beams with longitudinal rebar.

### 3.2. Experimental Setup

**Figure 6:**
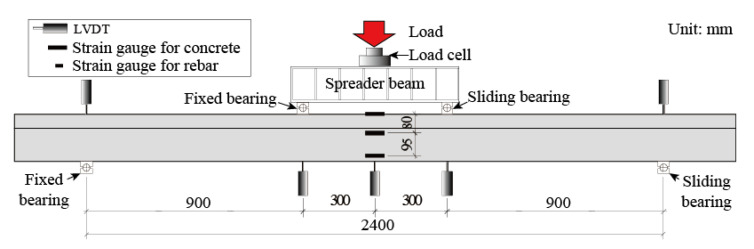
Load pattern and location of measuring instruments for load and displacement.

**Figure 7:**
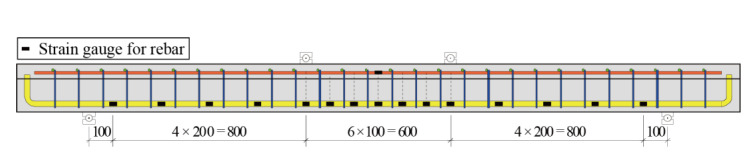
Locations of strain gauges on the rebar.

**Figure 8:**
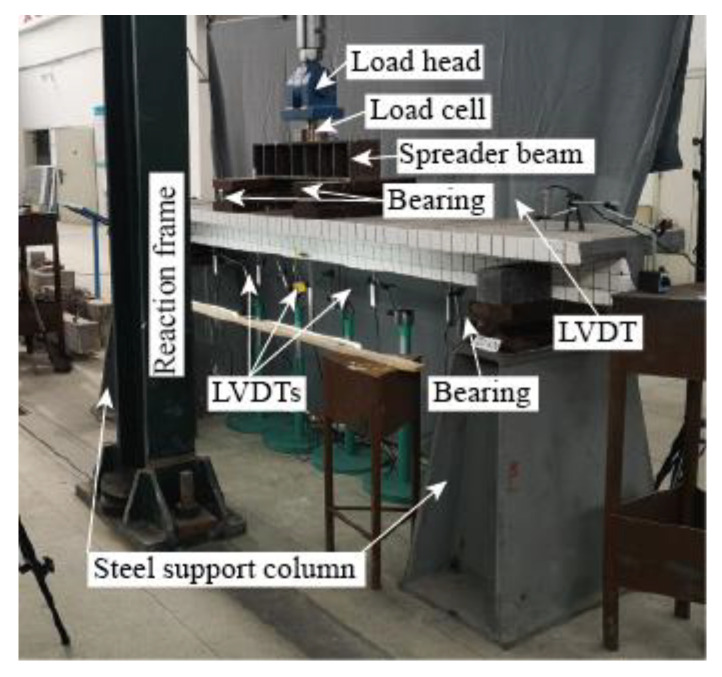
Experimental setup.

All T-shaped UHPC beams were tested under four-point loading, as shown in Figure 6. A monotonic load at a speed of 0.2 kN/s, which was provided by a closed-loop controlled hydraulic actuator (LETRY, Xian, Shaanxi Province, China), was applied at the center of the spreader beam. Two bearings were set on the flange of beams and under the spreader beam with a distance of 600 mm, while another two were set under the beam with a distance of 2400 mm. The dimensions of load bearings and support bearing are the same, and are 200 mm × 500 mm × 200 mm. Two load bearings were directly sited on the flange of beams while the two greased Teflon sheets were placed on the contact surface between the beam and one support bearing to act as a sliding bearing. A total of five LVDTs, including two LVDTs at the support bearings and the rest of three LVDTs at the locations as illustrated by Figure 6, were implemented to monitor the deflection. One load cell was placed between the load head and the spreader beam to monitor the applied load. Furthermore, as shown in Figure 7, a number of electrical strain gauges were used in each beam to monitor the strain data at different locations in steel bars. The experimental setup is shown in Figure 8.

## 4. Experimental Results and Discussion

### 4.1. Cracking Pattern and Failure Process

**Figure 9:**
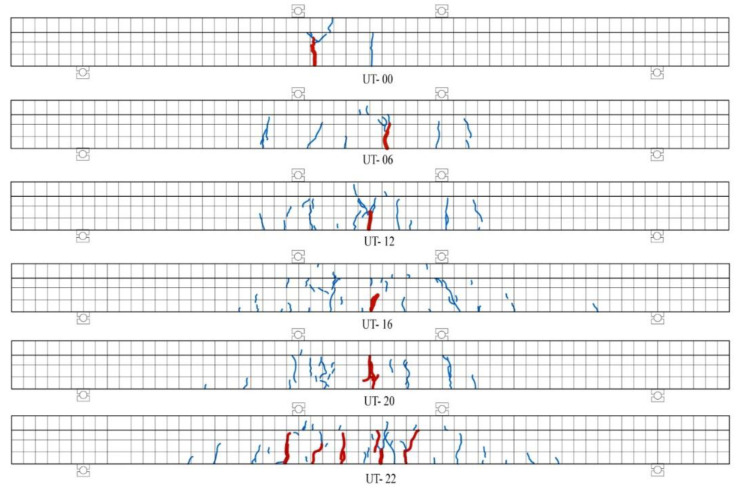
Cracking pattern of all beams after loading tests.

All the T-shaped UHPC beams exhibited elastic, cracking, and yielding phases, which were also typical flexural behaviors of properly reinforced concrete beams. At the beginning of the loading tests, the load of all the beams was linear to mid-span deflection. When they were loaded to the cracking flexural capacities, the first flexural crack was developed in the mid-span. As the loading progressed, more and more cracks were developed and propagated to the top of the beams in the pure flexural zone. Additionally, a sound similar to tearing cloth could be heard during the loading tests, because of the pulling off of steel fibers. Before the yielding of longitudinal bar, the crushing of the outermost fibers in flanges was not observed in the beams with longitudinal reinforcement. As for the beam without a longitudinal bar (UT-00), few cracks were developed in the pure flexural zone, and propagated into the flange. Consequently, the outmost UHPC in the flange was crushed and significant deflection occurred. Figure 9 shows the cracking pattern of all the beams after the loading tests, in which blue and red bold lines represent normal flexural cracks and localized cracks, respectively. As the longitudinal reinforcement ratio increased, more and more flexural cracks developed and distributed along the span. As for the T-shaped UHPC beam without longitudinal reinforcement (UT-00), few cracks were developed and localized, leading to flexural failure.

### 4.2. Load vs. Mid-Span Deflection Curves

**Figure 10:**
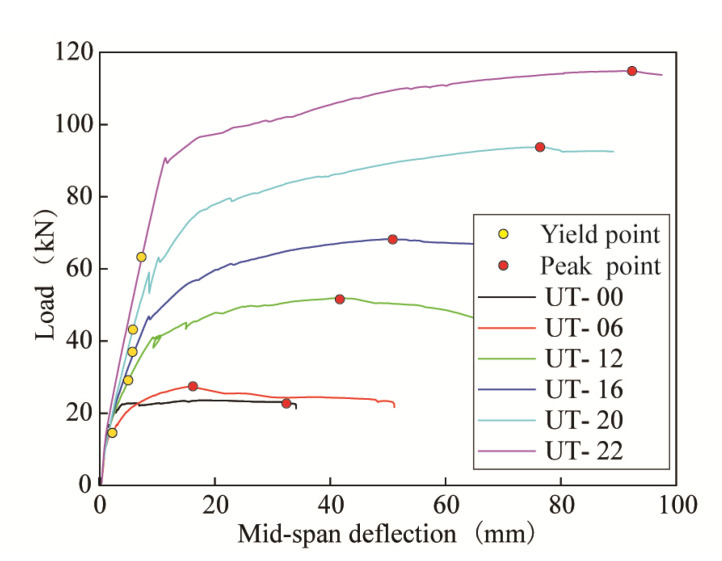
Load vs. mid-span deflection curves.

**Figure 11:**
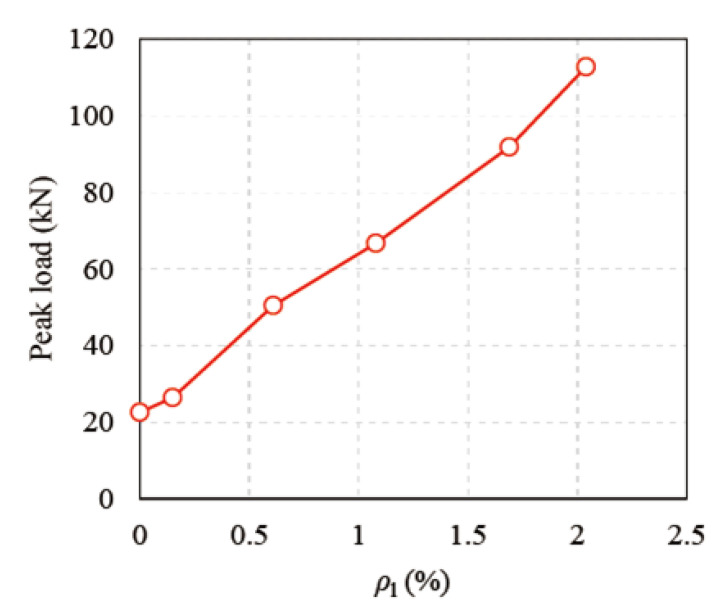
*ρ*_l_ vs. peak loads of all beams.

Based on the load vs. mid-span deflection curves of all the T-shaped UHPC beams, as shown in Figure 10, all the beams exhibited obvious ductile behavior. In the RC design theory, the minimum longitudinal reinforcement ratio is required to prevent the sudden failure of RC structural members, due to the tensile stress in concrete being transferred to the longitudinal rebar at the moment of concrete cracking. This principle is determined by the brittle nature of concrete. Different from RC structural members, UT-00 still exhibited ductile behavior, even without longitudinal rebar, attributing to the ductile tensile stress–strain relationship of UHPC. It also manifests that the principle of the minimum longitudinal reinforcement ratio in the RC design may not be applicable to UHPC structural members, but the longitudinal reinforcement ratio increases the dispersion of cracks and limits the localization of cracks. As for the rest of the beams with longitudinal rebar, the load and deformation capacities increased as the longitudinal reinforcement ratios increased. Figure 11 shows the relationship between the peak loads of the beams and the longitudinal reinforcement ratios (*ρ*_l_). It manifests that the peak load is almost linear to the longitudinal reinforcement ratio.

## 5. Theoretical Studies on Ultimate Flexural Capacity

To further study the flexural behavior of T-shaped UHPC beams with varying longitudinal reinforcement ratios, a section analysis was conducted to correlate with the experimental results and clarify the effects of parameters in constitutive models of UHPC.

### 5.1. Basic Assumptions

**Figure 12:**
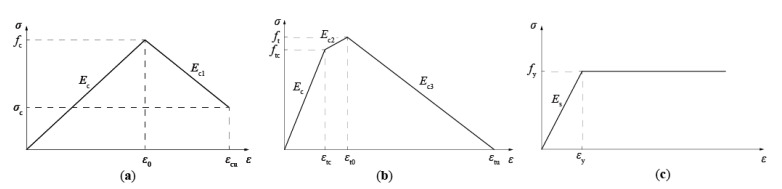
Material models. (**a**) Compression model of UHPC; (**b**) tensile model of UHPC; (**c**) model of steel rebar.

The following assumptions were determined to predict the flexural capacity of T-shaped UHPC beams:

1. The plane of the cross section remains plane after flexural deformation.
2. The compression model of UHPC is formulated by Equation (2), as illustrated by Figure 12a:
  (2) $$\sigma_{c}=\left\{\begin{array}{c} E_{c}\varepsilon_{c}\;\;\;\;\;\;\;\;\;\;\;\;\;\;\left(\varepsilon_{c}\leq \varepsilon_{0}\right)\\ f_{c}-E_{c1}\left(\varepsilon_{c}-\varepsilon_{0}\right)\;\;\left(\varepsilon_{0}<\varepsilon_{c}\leq \varepsilon_{cu}\right) \end{array}\right.$$
  where *σ_c_* and *ε_c_* are the compressive stress and strain, respectively; *f_c_* is the axial compressive strength, which is 141.8 MPa; *ε*_0_ is the strain corresponding to the axial compressive strength, which is 0.0027 in this study; *ε_cu_* is the ultimate compressive strain of UHPC, which is 0.0055 in this study [29]; and *E_c_* and *E_c_*_1_ are the moduli of the ascending and descending branches, and are 5.26 × 10^4^ MPa and 2.98 × 10^4^ MPa, respectively.
3. Based on the tensile test results of UHPC, the simplified tension model of UHPC adopted Equation (3), as shown by Figure 12b.
  (3) $$\sigma_{t}=\left\{\begin{array}{c} E_{c}\varepsilon_{t}\;\;\;\;\;\;\;\;\;\;\;\;\;\;\;\;\;\left(\varepsilon_{t}\leq \varepsilon_{tc}\right)\\ f_{tc}+E_{c2}\left(\varepsilon_{t}-\varepsilon_{tc}\right)\;\;\;\left(\varepsilon_{tc}<\varepsilon_{t}\leq \varepsilon_{t0}\right)\\ f_{t}-E_{c3}\left(\varepsilon_{t}-\varepsilon_{t0}\right)\;\;\;\;\;\left(\varepsilon_{t0}<\varepsilon_{t}\leq \varepsilon_{tu}\right) \end{array}\right.$$
  where *σ_t_* and *ε_t_* are the tensile stress and strain, respectively; *f_tc_* is the tensile cracking stress, which is 4.12 MPa in this study; *ε_tc_* is the tensile cracking strain, which is 0.00008; *f_t_* is the ultimate tensile strength, which is 8.42 MPa in this study, according to the UHPC tensile tests; *ε_tp_* is the first cracking strain; *ε_t_*_0_ is the strain corresponding to the ultimate tensile strength, which is 0.007 in this study, according to the tensile test results; *ε_tu_* is the ultimate tensile strain of UHPC, which is 0.05; and *E_c_*, *E_c_*_2_, and *E_c_*_3_ are the moduli of elasticity, and ascending and descending branches, which are 5.26 × 10^4^ MPa, 0.69 × 10^4^ MPa, and 0.17 × 10^4^ MPa, respectively.
4. The constitutive model of the steel bar adopted the bi-linear model [30], as shown in Figure 12c, and can be expressed by Equation (4), as follows:
  (4) $$\sigma_{s}=\left\{\begin{array}{c} E_{s}\varepsilon_{s}\;\;\;\;\left(\varepsilon_{s}\leq \varepsilon_{y}\right)\\ f_{y}\;\;\;\;\;\;\;\left(\varepsilon_{y}\leq \varepsilon_{s}\leq 0.01\right) \end{array}\right.$$
  where *σ_s_* and *ε_s_* are the stress and strain in steel rebar, respectively; *E_s_* is the elastic modulus of steel rebar, which is 2 × 10^5^ MPa in this study; and *f_y_* and *ε_y_* are the yield strength and strain of rebar, respectively.

### 5.2. Definition of Ultimate Limit State

**Figure 13:**
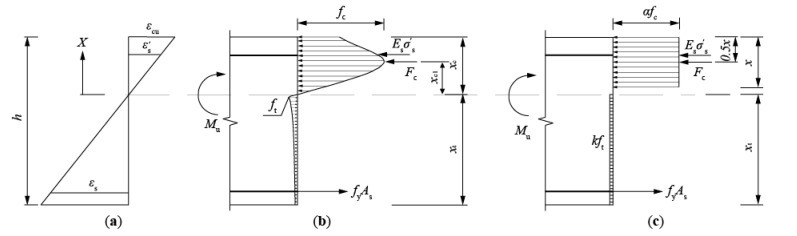
Ultimate limit state of T-shaped UHPC beam. (**a**) Distribution of strain; (**b**) distribution of actual stress; (**c**) equivalent stress block.

Based on the experimental phenomenon, as observed during the beam tests, the ultimate limit state of the flexure-dominated UHPC T-shaped beam was defined as that the longitudinal bar in tension was yielded and the outmost compression fiber of UHPC was crushed, as illustrated in Figure 10. The distribution of actual stress along the beam height is shown in Figure 13b.

### 5.3. Equivalent Stress Block in Tension and Compression

For simplicity of calculation, the distribution of compressive and tensile stress can be converted into equivalent stress block, based on integration, so that the resultant force and the location of the action point of the resultant force remain the same. As shown in Figure 13c, the resultant force of compression (*F_c_*) can be express by Equation (5), as follows:

(5) $$F_{c}=\int_{0}^{x_{c}}\sigma_{c}\left(\varepsilon_{c}\right)b\left(x\right)dx$$

where *σ_c_*(*ε_c_*) is the compressive stress in the compression zone, which is the function of compressive strain (*ε_c_*); and *b*(*x*) is the function of the height (*x*) to the neutral axis. In this study, the neutral axis is at the level of the flange, so that *b*(*x*) could be a constant of *b_f_*. The height from the action point of the resultant force of compression and the neutral axis (*x_c_*_1_) could be derived by Equation (6), as follows:

(6) $$x_{c1}=\frac{\int_{0}^{x_{c}}\sigma_{c}\left(\varepsilon_{c}\right)b_{f}xdx}{F_{c}}$$

The compressive strain (*ε_c_*) at the arbitrary height of *x* on the neutral axis in the compression side could be expressed by the following equation:

(7) $$\varepsilon_{c}=\kappa x$$

where *κ* is a proportional constant. The strain of the outmost fiber on the compression side is the ultimate compressive strain of *ε_cu_*. Therefore, Equation (8) could be expressed as follows:

(8) $$\varepsilon_{cu}=\kappa x_{c}$$

Based on Equations (7) and (8), the arbitrary height to the neutral axis (*x*) could be calculated as Equation (9), as follows:

(9) $$x=\varepsilon_{c}\frac{x_{c}}{\varepsilon_{cu}}$$

By differentiating Equation (9) with respect to *ε_c_*, it could be derived as Equation (10), as follows:

(10) $$dx=\frac{x_{c}}{\varepsilon_{cu}}d\varepsilon_{c}$$

By substituting Equation (10) into Equation (5), *F_c_* could be rewritten as Equation (11), as follows:

(11) $$F_{c}=b_{f}\frac{x_{c}}{\varepsilon_{cu}}\int_{0}^{\varepsilon_{cu}}\sigma_{c}\left(\varepsilon_{c}\right)d\varepsilon_{c}$$

Therefore, Equation (6) could be converted to Equation (12), based on Equation (11).

(12) $$x_{c1}=\frac{b_{f}{\left(\frac{x_{c}}{\varepsilon_{cu}}\right)}^{2}\int_{0}^{\varepsilon_{cu}}\sigma_{c}\left(\varepsilon_{c}\right)\varepsilon_{c}d\varepsilon_{c}}{F_{c}}$$

Based on dimensional analysis, Equations (11) and (12) could be converted in to Equations (13) and (14), as follows:

(13) $$k_{1}f_{c}=\frac{1}{\varepsilon_{cu}}\int_{0}^{\varepsilon_{cu}}\sigma_{c}\left(\varepsilon_{c}\right)d\varepsilon_{c}$$

(14) $$k_{2}=\frac{\int_{0}^{\varepsilon_{cu}}\sigma_{c}\left(\varepsilon_{c}\right)d\varepsilon_{c}}{\varepsilon_{cu}\int_{0}^{\varepsilon_{cu}}\sigma_{c}\left(\varepsilon_{c}\right)d\varepsilon_{c}}$$

where *k*_1_ and *k*_2_ are constants; and *f_c_* is the axial compressive strength. Then, Equations (11) and (12) could be simplified into Equations (15) and (16), as follows:

(15) $$F_{c}=b_{f}x_{c}k_{1}f_{c}$$

(16) $$x_{c1}=x_{c}k_{2}$$

As shown in Figure 13c, the bending moment, resulting from the compression, could be formulated by the following equation:

(17) $$M_{c}=b_{f}x_{c}k_{1}f_{c}\left(x_{c}k_{2}\right)=\alpha f_{c}b_{f}x\left(x_{c}-\frac{x}{2}\right)$$

where *x* is the total height of the equivalent compression block; *α* is the ratio of the equivalent stress to *f_c_*; and *β* is the ratio of the height of the equivalent stress block to the actual height of the compression zone. Then, *α* and *β* could be formulated by Equation (18) and (19), based on Equation (17).

(18) $$\alpha =\frac{k_{1}}{\beta }=\frac{k_{1}}{2\left(1-k_{2}\right)}$$

(19) $$\beta =\frac{x}{x_{c}}=2\left(1-k_{2}\right)$$

Based on the constitutive model of UHPC under compression, *α* and *β* were adopted as 0.82 and 0.83, respectively.

As for the equivalent stress block under tension, a reduction factor of *k* was induced, to simplify the actual tension distribution to the equivalent stress block. Then, the resultant force under tension (*F_t_*) could be formulated by Equation (20).

(20) $$F_{t}=kf_{t}b_{w}h_{w}+kf_{t}b_{f}\left(h_{f}-\frac{x}{\beta }\right)$$

where *k* is the reduction factor to the ultimate tensile strength of UHPC; *f_t_* is the ultimate tensile strength; *b_w_* is the width of the web; *h_w_* is the height of the web; *b_f_* is the width of the flange; and *h*_f_ is the height of the flange.

### 5.4. Predictive Equations for Flexural Capacity of T-Shaped UHPC Beams

As shown in Figure 10, Equation (21) could be formulated based on the equilibrium of internal forces acting on the cross section.

(21) $$E_{s}\varepsilon_{s}^{\prime }A_{s}^{\prime }+\alpha f_{c}b_{f}x=kf_{t}b_{w}h_{w}+kf_{t}b_{f}\left(h_{f}-\frac{x}{\beta }\right)+f_{y}A_{s}$$

where *ε_s_*′ is the compressive strain of rebar in compression side; *A_s_*′ is the total area of rebar on the compression side; and *A_s_* is the total area of rebar under tension. Based on the strain distribution as shown in Figure 13a, it could be formulated by Equation (22), as follows:

(22) $$\varepsilon_{s}^{\prime }=\frac{x_{c}-a_{s}^{\prime }}{x_{c}}$$

where *a_s_*′ is the height of the centroid of rebar under compression to the outmost fiber of UHPC under compression; *x_c_* is the actual height of the compression zone; *ε_cu_* is the ultimate compressive strain of UHPC, and is 0.0055 in this study. By substituting Equation (22) into (21), a quadratic equation about *x* could be obtained, formulated as Equation (23), as follows:

(23) $$A\cdot x^{2}+B\cdot x+C=0$$

in which *A*, *B* and *C* are parameters, which are expressed by Equation (24), as follows:

(24) $$\left\{\begin{array}{c} A=\frac{\alpha f_{c}b_{f}}{\beta }+\frac{kf_{t}b_{f}}{\beta^{2}}\\ B=\frac{1}{\beta }\left(E_{s}\varepsilon_{cu}A_{s}^{\prime }-f_{y}A_{s}-kf_{t}b_{w}h_{w}-kf_{t}b_{f}h_{f}\right)\\ C=-E_{s}\varepsilon_{cu}A_{s}^{\prime }a_{s}^{\prime } \end{array}\right.$$

By solving the quadratic equation about *x*, the height of the compressive equivalent stress block could be determined, and then the ultimate flexural capacity of T-shaped UHPC beams (*M_u_*) can be calculated by Equation (25), as follows:

(25) $$\begin{array}{c} M_{u}=\alpha f_{c}b_{f}\beta \left(\frac{x}{\beta }-\frac{x}{2}\right)+E_{s}\varepsilon_{s}^{\prime }A_{s}^{\prime }\left(a_{s}^{\prime }-\frac{x}{\beta }\right)+f_{y}A_{s}\left(h-a_{s}^{\prime }-\frac{x}{\beta }\right)\\ \;\;\;\;\;\;+kf_{t}b_{w}h_{w}\left(\frac{h_{w}}{2}+h_{f}-a_{s}^{\prime }\right)+kf_{t}b_{f}\left(h_{f}-\frac{x}{\beta }\right)\left(\frac{\beta h_{f}-x}{2\beta }\right) \end{array}$$

**Figure 14:**
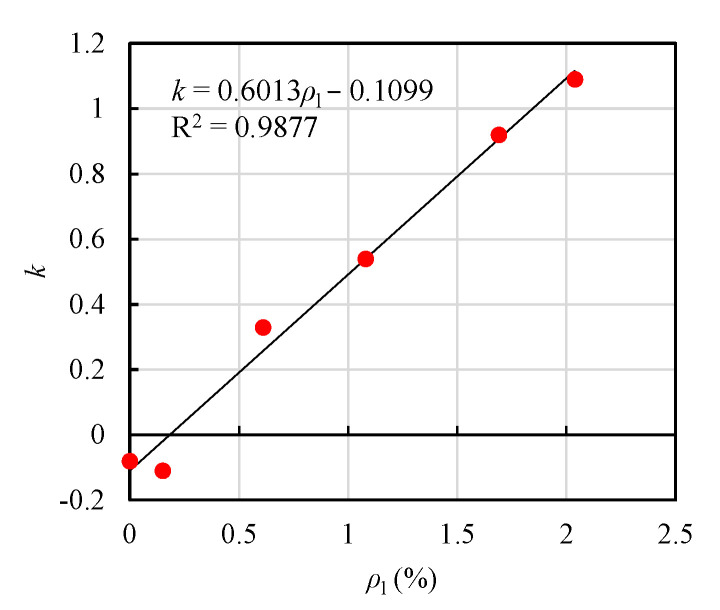
*k* versus *ρ*_l_.

In order to calculate *M_u_*, an unknown factor of *k* should be determined in advance. In this study, the value of *k* was determined by reverse calculation from the experimental results. For different T-shaped UHPC beams, with different longitudinal reinforcement ratios, the value of *k* varies linearly with the longitudinal reinforcement ratio (*ρ*_l_), as tabulated in Table 7, and shown in Figure 14. Hence, the reduction factor of *k* was fitted to be a linear equation about the longitudinal reinforcement ratio (*ρ*_l_), as expressed by Equation (26), as follows:

(26) $$k=0.6013\rho_{l}-0.1099$$

**Figure 15:**
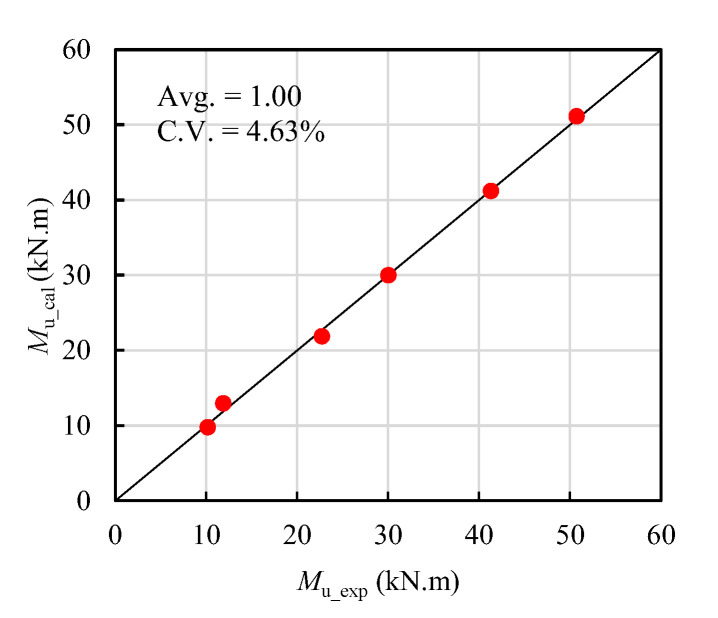
*M*_u_cal_ versus *M*_u_exp_ in this study.

Then, the predicted ultimate flexural capacity of T-shaped UHPC beams (*M*_u_cal_) could be calculated based on Equation (25) and (26). *M*_u_cal_, as well as the experimental ultimate flexural capacity (*M*_u_exp_) in this study, are tabulated in Table 7, and Figure 15 compares the *M*_u_cal_ and *M*_u_exp_ of all the beams in this study. The average value of the *M*_u_cal_-to-*M*_u_exp_ ratio is 1.00, and the coefficient of variation of the *M*_u_cal_-to-*M*_u_exp_ ratio is 4.63%, manifesting that the proposed equations agree well with the experimental results.

### 5.5. Validity of Proposed Equations

**Figure 16:**
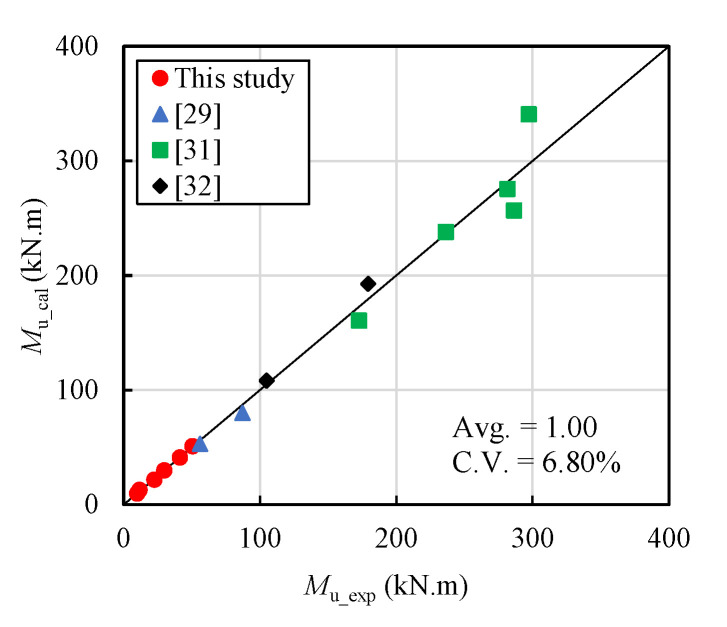
Validity of equations.

The existing experimental results about T-shaped UHPC beams [29,31,32] were employed to verify the validity of the proposed equations. The key parameters, and experimental and predicated ultimate flexural capacity of nine T-shaped UHPC beams are summarized in Table 8. Figure 16 compares the *M*_u_cal_ and *M*_u_exp_ of these nine beams in previous studies [30,31,32], as well as six beams in this study. It indicates that the proposed equations exhibit high accuracy for predicting the ultimate flexural capacity of T-shaped UHPC beams.

## 6. Discussion

The effect of reinforcement ratio, fiber content, and strength grade on the flexural behavior of UHPC beams with a rectangular section has been extensively studied [22,23,24,25,26,27]. However, a few studies on the flexural behavior of UHPC beams with a T-shaped section were carried out. A drawback of the existing study is the limited specimens and insufficiency in investigating the effect of the longitudinal reinforcement ratio on the flexural behavior of T-shaped UHPC beams, which results in unsatisfied accuracy for the existing predictive equations of the flexural capacity of T-shaped UHPC beams.

Based on the existing studies, tension and compression tests were performed on the UHPC, incorporating steel fiber with a 2% volume content. The tensile test results show that the tensile cracking and ultimate tensile strength of the UHPC adopted in this study are 4.14 and 8.42 MPa, respectively. After the first cracking, the tensile strain-hardening behavior was started, and continued to the ultimate tensile strength. Based on the tensile stress–strain curves from the tensile tests, it is found that the strain corresponding to the ultimate tensile strength is 0.007, which agrees well with the value described by the American design code [9]. The cubic and axial compressive strengths were obtained via cube and prism compression tests, and are 166.0 and 141.8 MPa, respectively. This indicates that the axial compressive strength of UHPC in this study is 85% of the cubic compressive strength, due to the end effect. The elastic modulus of UHPC in this study is 5.2 × 10^4^ MPa, which is higher than that of high-strength concrete.

To investigate the effect of the longitudinal reinforcement ratio on the flexural behavior of T-shaped UHPC beams, a total of six beams, including one without longitudinal reinforcement (UT-00) and the rest of five with varying longitudinal reinforcement ratios, were designed and fabricated. UT-00 and another beam with a longitudinal rebar with a 6 mm diameter (UT-06) was used to investigate whether shear tension failure exists in the UHPC beams. The experimental results revealed that two beams exhibited obvious yield points and plateau stages, which were similar to the properly reinforced concrete beam. This indicates that, different from the design theory of the RC structural member, the low limit for longitudinal reinforcement in T-shaped UHPC beams may not exist. As the longitudinal reinforcement ratio increases, the number of cracks, the yield, and the ultimate load of the beams increases. Further, the ultimate flexural capacity of six beams is almost linear to the longitudinal reinforcement ratio, which is also in accordance with the design theory of the RC structural member.

Sectional analysis was employed to predict the flexural capacity of T-shaped UHPC beams, by developing constitutive models of UHPC under tension and compression. The constitutive model of UHPC under tension adopted a tri-linear model, by considering the strain-hardening effect that was observed in the tensile tests. The material model of UHPC in compression adopted bi-linear models, which was also widely used in the existing research [22,23,29]. Based on the reverse calculation from the experimental results, it was found that the tension carried by UHPC varies from the longitudinal reinforcement ratio, and the reduction factor to the tensile strength of UHPC was formulated to be linear to the longitudinal reinforcement ratio. This indicates that the tension carried by UHPC decreases as the longitudinal reinforcement ratio increases, because the tension carried by rebar increases as the longitudinal reinforcement ratio increases. By taking the relationship between the reduction factor and the longitudinal reinforcement ratio into consideration, the predictive equations for the ultimate flexural capacity of T-shaped UHPC beams were proposed, and agreed well with the experimental result in this study and previous studies [29,31,32]. The other constitutive models of UHPC, with different linearity, have not been used to verify the validity of the proposed equation in this study; this could be the objective of a future study.

## 7. Conclusions

This study presents an experimental and theoretical study on the flexural behavior of T-shaped UHPC beams with varying longitudinal reinforcement ratios. Based on the experimental results and proposed theoretical equations, the following conclusions could be drawn:

(1) The tensile stress–strain relationship and compressive properties of UHPC were obtained based on uniaxial tension and compression tests. The strain-hardening behavior under tension was exhibited, and the cracking and ultimate tensile strength of UHPC were 4.14 and 8.42 MPa, respectively. The strain corresponding to the ultimate tensile strength of UHPC was 0.007. The axial compressive strength was 85% of the cubic compressive strength.
(2) Six T-shaped UHPC beams exhibited similar flexural behavior to that of properly reinforced concrete beams—elastic, cracking, and yielding phases. As the longitudinal reinforcement ratio increased, the number of cracks and load-carrying capacity also increased. The localization of cracks in T-shaped UHPC beams with low reinforcement ratios became more and more significant. As for the T-shaped beam without longitudinal reinforcement, it also exhibited obvious ductile behavior, indicating that the principle of the minimum reinforcement ratio in the reinforced concrete design may not be applicable to UHPC structural members, but the longitudinal reinforcement ratio increases the dispersion of cracks and limits the localization of cracks.
(3) Based on the assumptions, and the simplified material model of UHPC under tension and compression, resulting from the material test results, the predicted equations for the ultimate flexural capacity of T-shaped UHPC beams were proposed, by inducing the reduction factor to the ultimate tensile strength of UHPC. It was found that the value of the reduction factor is almost linear to the longitudinal reinforcement ratio. By comparing with the experimental results in this study and previous studies, the proposed equations agree well with the experiments, indicating good validation.

## Figures and Tables

**Table 1:** Properties of steel fibers.

Length (mm)	Diameter (mm)	Strength (MPa)	Shape	Surface
13	0.2	>2850	Straight	Smooth

**Table 2:** Mix proportion of UHPC.

W/B ^1^ (%)	Unit Weight (kg/m^3^)				
	Water	Binder	Quartz Sand	Steel Fiber	Superplastizer ^2^
18	164.51	913.96	1096.75	158	4.57

^1^ W/B stands for water to binder ratio; ^2^ polycarboxylic acid superplasticizer (Model: 3301C, provided by Sika AG company).

**Table 3:** Compressive properties of UHPC.

Material	Cubic Compressive Strength		Axial Compressive Strength		Elastic Modulus	
	Mean ^1^ (MPa)	C.V. ^2^ (%)	Mean ^1^ (MPa)	C.V. ^2^ (%)	Mean ^1^ (MPa)	C.V. ^2^ (%)
UHPC	166.0	4.0	141.8	4.2	5.2 × 10^4^	1.1

^1^ Mean stands for mean value of samples; ^2^ C.V. stands for the coefficient of variation.

**Table 4:** Tensile properties of UHPC.

Material	First Cracking Strength		Ultimate Tensile Strength (MPa)		Strain Corresponding to the Ultimate Tensile Strength	
	Mean ^1^ (MPa)	C.V. ^2^ (%)	Mean ^1^ (MPa)	C.V. ^2^ (%)	Mean ^1^ (MPa)	C.V. ^2^ (%)
UHPC	4.14	1.2	8.42	7.4	0.007	8.9

^1^ Mean stands for mean value of samples; ^2^ C.V. stands for the coefficient of variation.

**Table 5:** Tensile test results of steel bars.

Diameter (mm)	*f*_y_ ^1^		*f*_t_ ^2^		Usage	Surface
	Mean ^3^ (MPa)	C.V. ^4^ (%)	Mean ^3^ (MPa)	C.V. ^4^ (%)		
22	470.5	0.2	651.0	0.0	Longitudinal tensile bar	Deformed
20	415.5	0.5	604.1	0.5		
16	429.5	0.3	618.6	0.3		
12	479.5	1.0	662.2	0.3		
6	529.7	0.3	537.0	0.3		
10	519.9	0.8	623.6	0.5	Steel in flange	

^1^*f*_y_ stands for tensile yield strength; ^2^ *f*_t_ stands for ultimate tensile strength; ^3^ mean stands for the mean value; ^4^ C.V. stands for coefficient of variation.

**Table 6:** Summary of beams.

Beam	*L* (mm)	*h* (mm)	*h*_0_ (mm)	*b*_f_ (mm)	*b*_w_*h* (mm^2^)	Longitudinal Bar	
						*A*_s_ (mm^2^)	*ρ*_l_ (%)
UT-00	3000	200	165	500	18,600	0	0
UT-06						28.27	0.15
UT-12						113.10	0.61
UT-16						201.06	1.08
UT-20						314.16	1.69
UT-22						380.13	2.04

**Table 7:** Comparison between predicted and experimental ultimate flexural capacity.

Beam	*x* (mm)	*k*	*M*_u_cal_ (kN.m)	*M*_u_exp_ (kN.m)	*M*_u_cal_/*M*_u_exp_
UT-00	6.91	−0.11	9.78	10.17	0.96
UT-06	7.28	−0.12	12.92	11.88	1.09
UT-12	8.48	0.26	21.84	22.73	0.96
UT-16	9.75	0.54	29.97	30.02	1.00
UT-20	11.60	0.91	41.18	41.31	1.00
UT-22	13.05	1.12	51.12	50.72	1.01

**Table 8:** Validation of proposed equations in previous studies.

Ref.	ID	*x* (mm)	*k*	*M_u_cal_* (kN.m)	*M_u_exp_* (kN.m)	*M_u_cal_*/*M_u_exp_*
[29]	B-S65-16	13.49	0.37	53.19	56.16	0.95
	B-S65-20	16.44	0.64	79.98	87.21	0.92
[31]	T-1	30.38	0.95	160.61	172.94	0.93
	T-2	44.23	1.64	238.17	236.43	1.01
	T-3	48.76	2.04	256.84	286.47	0.90
	T-4	59.73	2.54	340.77	297.32	1.15
	T-5	48.18	1.64	275.65	281.61	0.98
[32]	T1	20.49	0.62	108.22	105.12	1.03
	T2	38.76	2.07	192.62	179.42	1.07

## Data Availability

The data presented in this study are available in the article.
